# Effect of semaglutide on COVID-19 and other infections: an analysis from the FLOW randomized clinical trial

**DOI:** 10.1093/ndt/gfag036

**Published:** 2026-02-23

**Authors:** Brian Rayner, Kenneth W Mahaffey, Johannes F E Mann, Richard E Pratley, Peter Rossing, Nicolas Belmar, Heidrun Bosch-Traberg, Ole Kleist Jeppesen, Minh Chau Tran, Gil Chernin, Katherine R Tuttle

**Affiliations:** Division of Nephrology and Hypertension, Groote Schuur Hospital and University of Cape Town, Cape Town, South Africa; Department of Medicine, Stanford Center for Clinical Research, Stanford University School of Medicine, Stanford, CA, USA; KfH Kidney Centre, München, Germany; University Hospital, Friedrich-Alexander University, Erlangen, Germany; AdventHealth Translational Research Institute, Orlando, FL, USA; Steno Diabetes Center Copenhagen, Herlev, Denmark; Department of Clinical Medicine, University of Copenhagen, Copenhagen, Denmark; Novo Nordisk A/S, Søborg, Denmark; Novo Nordisk A/S, Søborg, Denmark; Novo Nordisk A/S, Søborg, Denmark; Novo Nordisk A/S, Søborg, Denmark; Kaplan Medical Center, Hebrew University of Jerusalem, Rehovot, Israel; Division of Nephrology, University of Washington School of Medicine, Seattle, WA, USA; Providence Medical Research Center, Providence Inland Northwest Health, Spokane, WA, USA

**Keywords:** CKD, clinical trial, diabetic kidney disease, pneumonia, type 2 diabetes

## Abstract

**Background:**

In the FLOW trial (Evaluate Renal Function with Semaglutide Once-Weekly; ClinicalTrials.gov, NCT03819153), once-weekly subcutaneous semaglutide 1.0 mg versus placebo reduced the risk of major kidney, cardiovascular (CV) and mortality outcomes in participants with type 2 diabetes (T2D) and chronic kidney disease (CKD). This pre-specified analysis assesses the impact of semaglutide on risks of serious infections and coronavirus disease 2019 (COVID-19) in the FLOW trial.

**Methods:**

The main outcome was a three-component composite [time from randomization to first infection serious adverse event (SAE), first hospitalization due to infection, or all-cause death]. Other outcomes included all infection SAEs, COVID-19 adverse events, COVID-19 SAEs, death due to infection and death concurrent with a COVID-19 SAE.

**Results:**

Semaglutide reduced the risk of the main outcome versus placebo [hazard ratio (HR) (95% confidence interval) 0.79 (0.69–0.89); *P* = .0002]. This risk reduction was greater in subgroups with baseline hemoglobin A_1c_ >8% [HR 0.63 (0.52–0.76)] versus ≤8% [HR 0.93 (0.79–1.11); *P_interaction_* = .0027], and urine albumin-to-creatinine ratio (UACR) ≥2000 mg/g [HR 0.53 (0.39–0.72)] versus UACR <100 mg/g [HR 0.90 (0.58–1.39); *P_interaction_* = .0367]. The semaglutide group had a lower rate of infection SAEs (17.9% versus 21.3%; *P* = .0070), COVID-19 SAEs (6.7% versus 8.8%; *P* = .0155), COVID-19 adverse events (20.3% versus 22.9%; *P* = .0190) and hospitalization due to infection (17.5% versus 20.4%; *P* = .015).

**Conclusions:**

Semaglutide reduced risks of infection SAEs, hospitalization due to infection, and all-cause death with greater benefit among those with poorer glycemic control and greater baseline albuminuria. These findings support potential clinical benefits of semaglutide beyond kidney, CV and metabolic outcomes in high-risk patients with T2D and CKD.

## Graphical Abstract

**Graphical Abstract:**
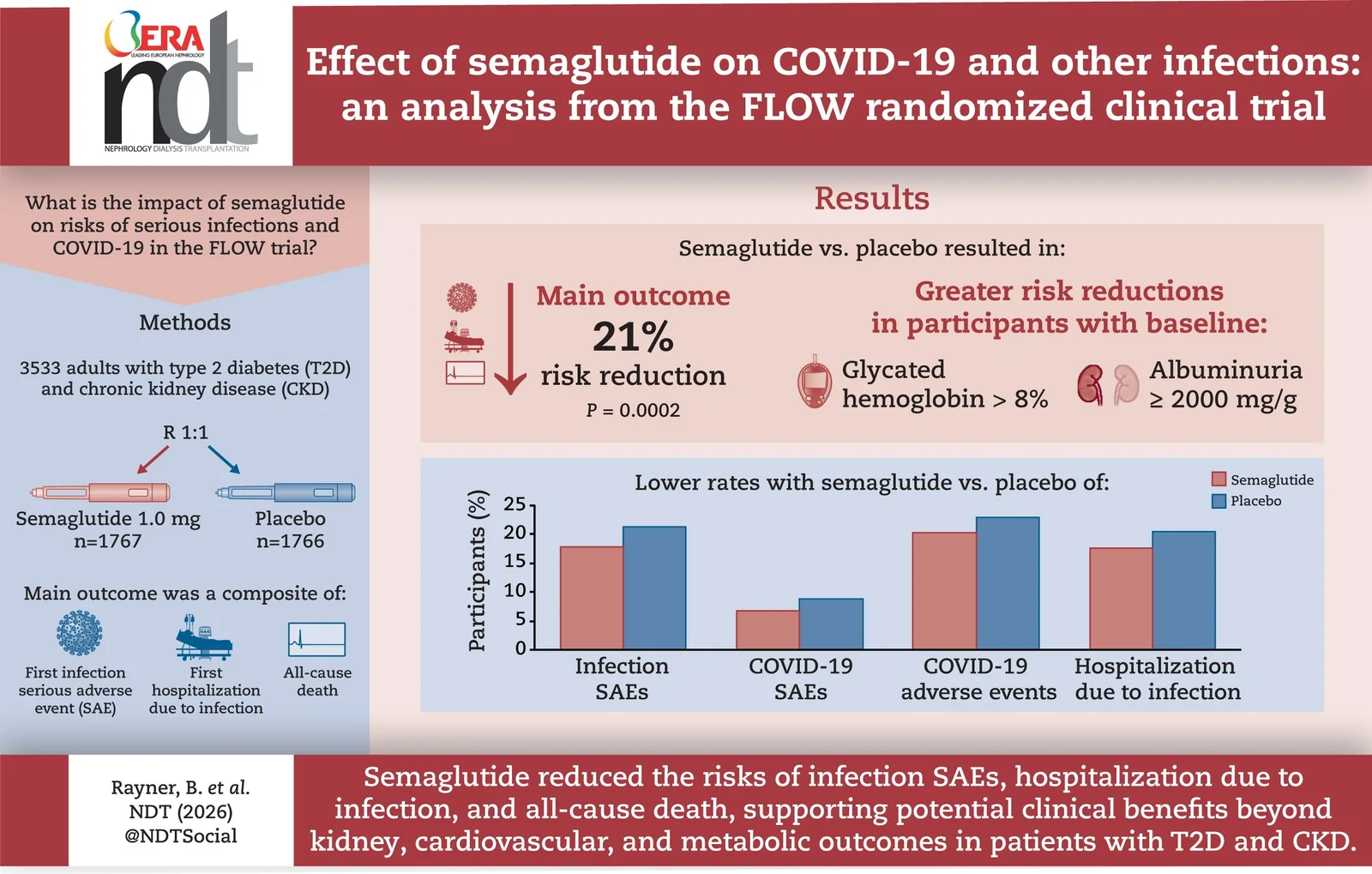

KEY LEARNING POINTS **What was known:**

- Approximately 40% of people with type 2 diabetes (T2D) develop chronic kidney disease (CKD). The co-occurrence of these conditions is associated with increased morbidity, including serious infections and coronavirus disease 2019 (COVID-19).
- People with T2D and CKD who develop infections represent an especially high-risk population who are more likely to progress to kidney failure, experience cardiovascular (CV) events or die.
- The FLOW phase 3 trial demonstrated the significant clinical benefit of semaglutide 1.0 mg once-weekly on major kidney, CV and mortality outcomes in people with T2D and CKD; however, the impact of semaglutide 1.0 mg on outcomes related to serious infections has not previously been explored in this population.

**This study adds:**

- The COVID-19 pandemic occurred during the FLOW phase 3 clinical trial, with the most severe phase (March 2020 to March 2022) fully overlapping with the trial period. This provided an opportunity to assess the effect of the COVID-19 pandemic in a population at high risk of serious complications.
- This secondary analysis demonstrated a reduced risk of infection serious adverse events (SAEs), hospitalization due to infection, or all-cause death with subcutaneous semaglutide 1.0 mg, relative to placebo; there was also a lower overall incidence of infection SAEs, COVID-19 adverse events and COVID-19 SAEs with semaglutide than with placebo.
- Significant interactions were observed between effects of semaglutide on risks of serious infections and high levels of hemoglobin A_1c_ or albuminuria at baseline, with greater benefit among those with poorer glycemic control or more severe CKD.

**Potential impact:**

- The present findings support a potential role for subcutaneous semaglutide 1.0 mg once-weekly in mitigating risks of serious infection and COVID-19 events in people with T2D and CKD, highlighting important clinical benefits beyond kidney, CV and metabolic outcomes.

## INTRODUCTION

Chronic kidney disease (CKD) affects approximately 10% of the world’s population and is a major cause of global mortality [1–3]. The prevalence of CKD is rising, primarily due to increased prevalence of diabetes, obesity and hypertension, as well as an aging population [4]. Notably, approximately 40% of people with type 2 diabetes (T2D) develop CKD [5, 6]. The co-occurrence of T2D and CKD is associated with considerable morbidity and mortality including cardiovascular (CV) disease (CVD), kidney failure and other severe complications, including infections [7–9].

People with T2D and CKD are at an increased risk of developing serious infections due to poor glycemic control, compromised immune function, comorbidities such as CVD, and frequent hospitalization with exposure to nosocomial infections [10–13]. Urinary tract, respiratory and skin infections are common [10]. Patients with infections are more likely to progress to kidney failure, experience CV events or die than those without, and thus represent an especially high-risk population [14]. Similarly, people with T2D and CKD are at high risk of developing coronavirus disease 2019 (COVID-19), particularly those with kidney failure treated by dialysis [15]. Moreover, underlying CKD or diabetes was associated with increased mortality from COVID-19 [16, 17].

In the FLOW (Evaluate Renal Function with Semaglutide Once-Weekly) phase 3 clinical trial (NCT03819153), treatment with once-weekly subcutaneous semaglutide 1.0 mg, a glucagon-like peptide-1 receptor agonist (GLP-1 RA), reduced risks of major kidney, CV and mortality outcomes in participants with T2D and CKD [18]. The global COVID-19 pandemic occurred during the conducting of the FLOW trial, with the outbreak declared a public health emergency in January 2020, during the first year of recruitment. The most severe phase of the pandemic (March 2020 to March 2022) fully overlapped with the trial period (June 2019 to January 2024). Following the onset of the pandemic, the trial protocol was amended, and local guidance was adjusted to mitigate risk of COVID-19 exposure to participants and staff. Measures were also taken to ensure the integrity of the clinical trial as well as participant recruitment and retention. The particular attention to infection management provided opportunity to assess impacts of the COVID-19 pandemic in a patient population at high risk of developing serious complications [19]. In this secondary pre-specified analysis, we evaluated the effects of once-weekly subcutaneous semaglutide 1.0 mg on the incidence of infection serious adverse events (SAEs), including COVID-19, as well as hospitalization due to infection and all-cause death.

## MATERIALS AND METHODS

### Participants and trial design

The FLOW trial design and primary results have been published previously [18, 19]. In brief, eligible participants had T2D [with glycated hemoglobin (HbA_1c_) levels ≤10%] and CKD [defined as an estimated glomerular filtration rate (eGFR) of 25–75 mL/min/1.73 m^2^ and urine albumin-to-creatinine ratio (UACR) >300 and <5000 if eGFR was ≥50 mL/min/1.73 m^2^, or UACR >100 and <5000 if eGFR was 25–<50 mL/min/1.73 m^2^]. Participants were randomized 1:1 to once-weekly subcutaneous semaglutide 1.0 mg or matching placebo from June 2019 to May 2021 with randomization stratified by baseline use of a sodium–glucose cotransporter-2 inhibitor (SGLT2i).

For reporting purposes, the start date of the global COVID-19 pandemic was determined to be 1 February 2020. A total of 1429 (40.4%) participants were randomized prior to this date. To mitigate the impact of the pandemic on the trial, alternative procedures were implemented, where permitted by local regulations. These included increased flexibility of visit schedules, visits at home by remote technologies or in-person visits from site staff, and alternative methods of access to the investigational product such as product pick-up from pharmacies or delivery by courier or site staff. Moreover, exclusion criteria were amended to allow for simultaneous participation in a preventive or interventional COVID-19 trial, if the last dose of the investigational product was received over 30 days before screening in the FLOW trial. Co-participation in COVID-19 trials was not allowed by sites in China due to country-specific regulations. Approved COVID-19 vaccines received at screening or during the trial were recorded in the electronic case report form. The trial protocol was also amended to implement the systematic collection of COVID-19 AEs.

The FLOW trial was conducted in accordance with the protocol, the Declaration of Helsinki and the International Council for Harmonisation of Technical Requirements for Registration of Pharmaceuticals for Human Use (ICH) Good Clinical Practice guidelines. All participants provided written informed consent prior to any trial-related activity. Trial data were reviewed and evaluated by an independent data monitoring committee (DMC) at predefined time points. Following a prespecified interim analysis in October 2023, the DMC recommended early trial completion.

### Outcomes

In this pre-specified secondary analysis, the main outcome was a composite comprising the time to the first infection SAE, first hospitalization due to infection or all-cause death. Additional outcomes in this analysis were the incidence of infection SAEs, COVID-19 adverse events (AEs), COVID-19 SAEs, hospitalization with a fatal outcome due to infection, death due to infection, all-cause death concurrent with a COVID-19 AE and all-cause death concurrent with an infection SAE. The primary outcome of the FLOW trial (composite of ≥50% reduction in eGFR; eGFR <15 mL/min/1.73 m^2^; initiation of chronic kidney replacement therapy; kidney death; or CV death) concurrent with COVID-19 was also assessed. Deaths were considered concurrent with a COVID-19 SAE if they occurred between the first day of the COVID-19 SAE and up to 30 days after either the end date of the COVID-19 SAE or the end of hospitalization with a COVID-19 SAE.

SAEs (including infection SAEs; see Supplementary data, Methods), AEs leading to treatment discontinuation and AEs of special interest were systematically collected by site investigators. All COVID-19-related AEs, irrespective of seriousness, were reported by the investigator by standard AE reporting; suspected COVID-19 was reported based on a typical clinical presentation irrespective of a positive COVID-19 test and in the absence of clinical symptoms a positive COVID-19 test. All deaths were adjudicated and subcategories for non-CV or non-kidney deaths were assigned, including death due to infection with or without COVID-19.

### Statistical analysis

Efficacy outcomes were analyzed according to the intention-to-treat principle using data from the in-trial period of all randomized participants regardless of treatment adherence or use of other medications. Hazard ratios (HRs) for the various study outcomes were analyzed using a Cox proportional hazards model with treatment as a categorical fixed factor, stratified by use of an SGLT2i (yes or no) at baseline, and for subgroup analyses, an interaction term between treatment and subgroup was added. Participants without events of interest were censored at the end of their in-trial observation period. Cumulative incidence estimates were based on the Aalen–Johansen estimator with all-cause death modeled as a competing risk when all-cause death was not part of the outcome. No adjustment for multiplicity or alpha-protection was performed, and two-sided *P*-values <.05 were considered significant. All statistical analyses were performed using SAS software version 9.4.

## RESULTS

### Participants

A total of 3533 participants were randomized (semaglutide: *n* = 1767; placebo: *n* = 1766). Randomization began on 26 June 2019; by 31 January 2020, 1429 (40.4%) of the total 3533 participants had been randomized. In both treatment groups, participants with an infection SAE or COVID-19 AE during the trial had higher baseline body weight and body mass index (BMI) and were more likely to have a history of prior myocardial infarction or stroke than those without an infection (Table 1). Out of all participants, 73.6% received at least one COVID-19 vaccination during the trial, with similar proportions between the semaglutide (74.5%) and placebo (72.7%) treatment groups. A similar proportion of participants who experienced a COVID-19 AE during the trial were vaccinated against COVID-19 compared with those who did not experience a COVID-19 AE (72.8% versus 73.6%, respectively); this was also observed by treatment group (75.1% versus 74.2% with semaglutide, and 70.8% versus 73.0% with placebo; Supplementary data, Tables S1 and S2). Of all important protocol deviations during the trial, 13.7% (241/1755) were related to COVID-19, with both treatment groups affected comparably.

**Table 1:** Baseline characteristics by the occurrence of an infection SAE or COVID-19 AE during the trial.

	Semaglutide 1.0 mg		Placebo	
Characteristic	Without infection/COVID-19 (*n* = 1244)	With infection/COVID-19 (*n* = 523)	Without infection/COVID-19 (*n* = 1182)	With infection/COVID-19 (*n* = 584)
History of CV event				
No prior MI or stroke	962 (77.3)	383 (73.2)	913 (77.2)	430 (73.6)
Prior MI or stroke	272 (21.9)	133 (25.4)	256 (21.7)	147 (25.2)
Sex				
Female	368 (29.6)	151 (28.9)	389 (32.9)	161 (27.6)
Male	876 (70.4)	372 (71.1)	793 (67.1)	423 (72.4)
Age, years	66.5 (9.0)	66.8 (9.1)	66.9 (9.0)	66.4 (8.9)
Ethnicity				
Hispanic or Latino	205 (16.5)	68 (13.0)	180 (15.2)	103 (17.6)
Not Hispanic or Latino	998 (80.2)	423 (80.9)	950 (80.4)	461 (78.9)
Not reported	41 (3.3)	32 (6.1)	52 (4.4)	20 (3.4)
Race				
American Indian or Alaska Native	7 (0.6)	2 (0.4)	12 (1.0)	4 (0.7)
Asian	309 (24.8)	130 (24.9)	302 (25.5)	105 (18.0)
Black or African American	56 (4.5)	22 (4.2)	59 (5.0)	23 (3.9)
Native Hawaiian or other Pacific Islander	2 (0.2)	0	3 (0.3)	0
Not reported	21 (1.7)	18 (3.4)	29 (2.5)	11 (1.9)
Other	34 (2.7)	11 (2.1)	27 (2.3)	23 (3.9)
White	815 (65.5)	340 (65.0)	750 (63.5)	418 (71.6)
Region				
Asia	328 (26.4)	150 (28.7)	306 (25.9)	128 (21.9)
Europe	336 (27.0)	136 (26.0)	332 (28.1)	159 (27.2)
North America	287 (23.1)	136 (26.0)	294 (24.9)	148 (25.3)
Other	293 (23.6)	101 (19.3)	250 (21.2)	149 (25.5)
Body weight, kg	88.87 (19.72)	90.87 (19.94)	88.09 (20.83)	93.28 (21.64)
BMI, kg/m^2^	31.73 (5.88)	32.39 (6.63)	31.57 (6.37)	32.88 (6.77)
HbA_1c_, %	7.79 (1.33)	7.81 (1.22)	7.67 (1.24)	7.96 (1.34)
eGFR, mL/min/1.73 m^2^	47.04 (15.77)	46.59 (15.26)	47.44 (14.48)	46.41 (15.01)
UACR, mg/g^a^	511.06 (215.40)	515.48 (212.29)	458.59 (198.15)	565.16 (214.54)
Systolic blood pressure, mmHg	138.2 (15.5)	140.4 (17.4)	137.7 (15.1)	139.7 (15.9)
Diastolic blood pressure, mmHg	77.0 (9.8)	76.1 (10.4)	75.9 (9.9)	76.4 (10.4)
Diabetes duration, years				
<15	564 (45.3)	210 (40.2)	526 (44.5)	227 (38.9)
≥15	679 (54.6)	313 (59.8)	656 (55.5)	357 (61.1)
Smoking status				
Current smoker	165 (13.3)	58 (11.1)	153 (12.9)	53 (9.1)
Never smoker	633 (50.9)	250 (47.8)	601 (50.8)	263 (45.0)
Previous smoker	446 (35.9)	215 (41.1)	428 (36.2)	268 (45.9)

Values are no. (%) or mean (standard deviation), unless otherwise specified.

a UACR values are geometric mean (coefficient of variation). MI, myocardial infarction.

### Outcomes and COVID-19 events

**Figure 1:**
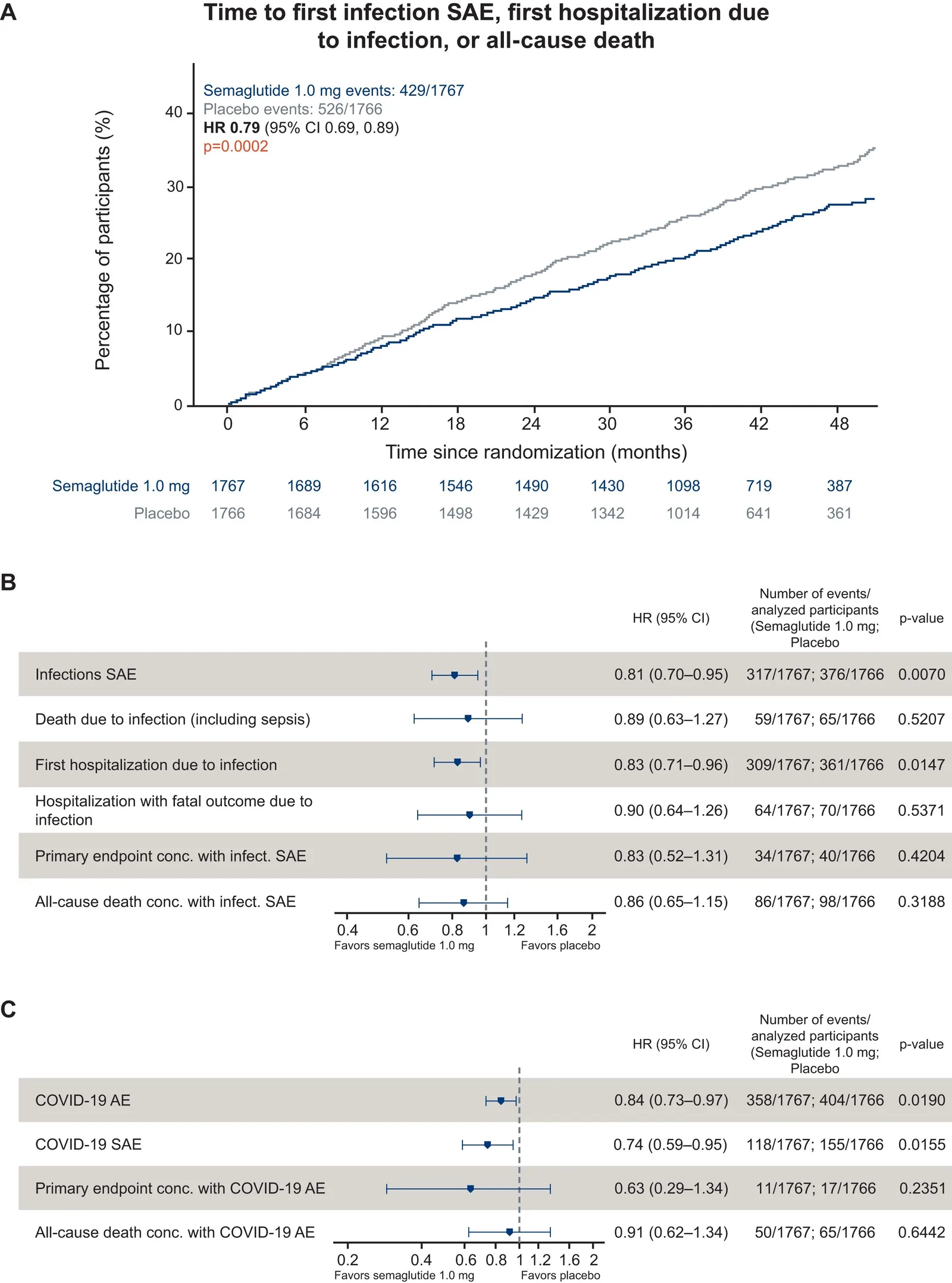
Effect of semaglutide on the main outcome and secondary infection, and COVID-19-related events. Data are from the in-trial observation period. (**A**) Time from randomization to first occurrence of the main study outcome (time to first infection SAE, first hospitalization due to infection or all-cause death). Numbers shown in the lower panel represent the number of participants at risk. (**B, C**) Forest plots showing time from randomization to (B) infection-related outcomes and (C) COVID-19-related outcomes. conc., concurrent; infect., infection.

Semaglutide treatment significantly reduced the risk of the main outcome, a composite of the first infection SAE, first hospitalization due to infection or all-cause death), with an HR [95% confidence interval (CI)] of 0.79 (0.69–0.89; *P* = .0002). There were 429 events in the semaglutide group versus 526 events in the placebo group (Fig. 1A).

**Figure 2:**
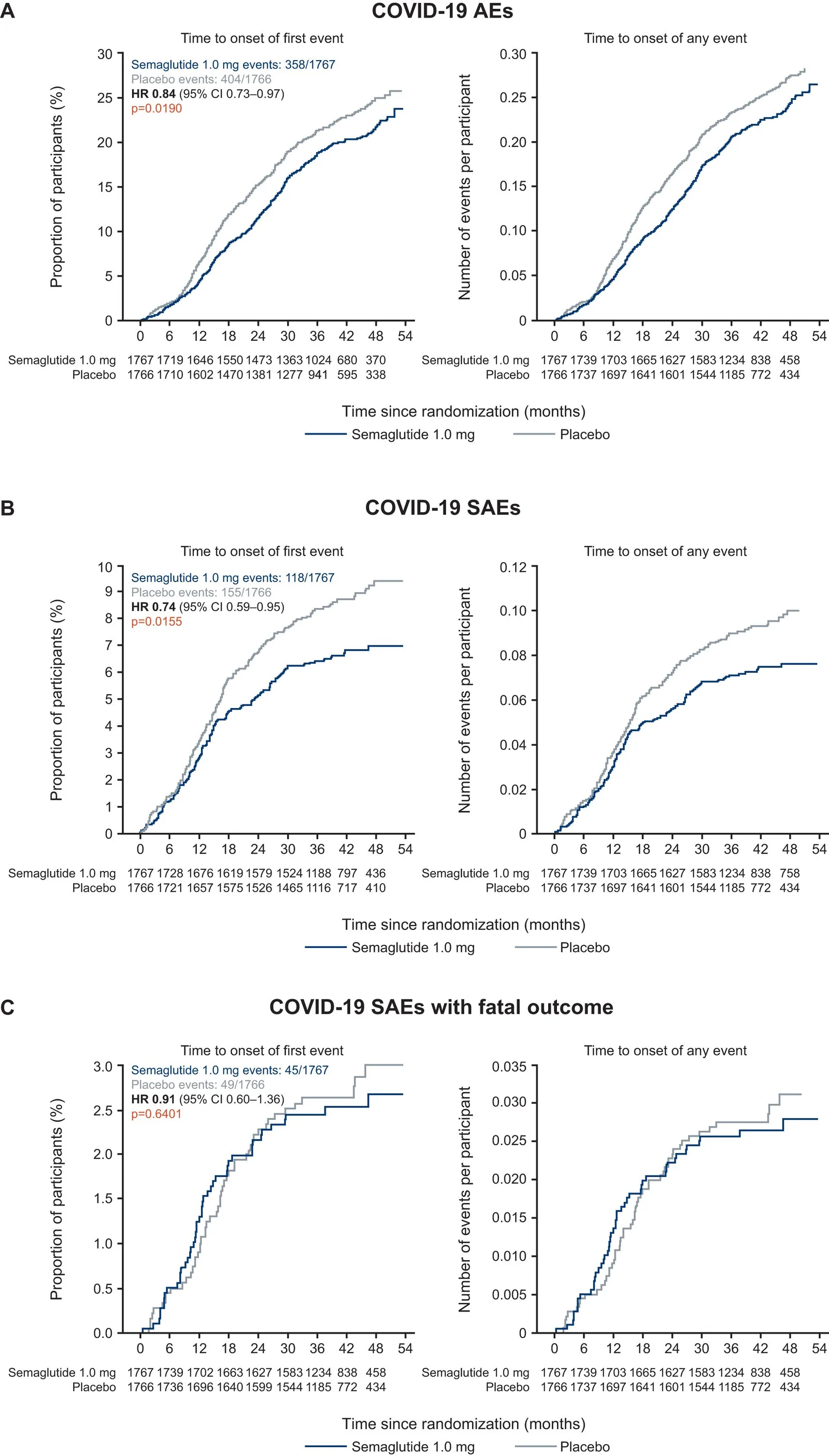
Time to first COVID-19 AEs, SAEs and SAEs with a fatal outcome. Data are from the in-trial observation period. Plots show time from randomization to first and any (**A**) COVID-19 AE, (**B**) COVID-19 SAE and (**C**) COVID-19 SAE with fatal outcome.

Smaller proportions of participants receiving semaglutide versus placebo, respectively, had an infection SAE (17.9% versus 21.3%), COVID-19 AE (20.3% versus 22.9%) or COVID-19 SAE (6.7% versus 8.8%) during the trial. This led to a reduction in risk of an infection SAE [317 versus 376 events; HR (95% CI) 0.81 (0.70–0.95); *P* = .0070], COVID-19 AE [358 versus 404 events; HR (95% CI) 0.84 (0.73–0.97); *P* = .0190) and a COVID-19 SAE [118 versus 155 events; HR (95% CI) 0.74 (0.59–0.95); *P* = .0155] with semaglutide versus placebo, respectively (Figs 1B, C, and 2A, B). The most common infection SAEs reported during the trial were COVID-19 pneumonia (3.9% versus 4.7%), other pneumonia (3.7% versus 4.2%) and COVID-19 infection (3.3% versus 4.2%), all with a lower incidence in the semaglutide group versus placebo, respectively (Supplementary data, Fig. S1).

Semaglutide treatment reduced the risk of hospitalization due to an infection during the trial period compared with placebo [309 versus 361 events; HR (95% CI) 0.83 (0.71–0.96); *P* = .0147]. There were no significant treatment differences in individual events of the primary FLOW outcome concurrent with a COVID-19 AE, all-cause death concurrent with a COVID-19 AE, death due to infection, hospitalization with a fatal outcome due to an infection or all-cause death concurrent with an infection SAE (Fig. 1B and C). Moreover, the incidence of serious COVID-19 events with a fatal outcome was similar between treatment groups (45 events with semaglutide versus 49 events with placebo; Fig. 2C). In both treatment groups, the frequency of acute kidney injury events reported was higher in participants with a COVID-19 AE than in those without (Supplementary data, Fig. S2).

### Subgroup analyses

**Figure 3:**
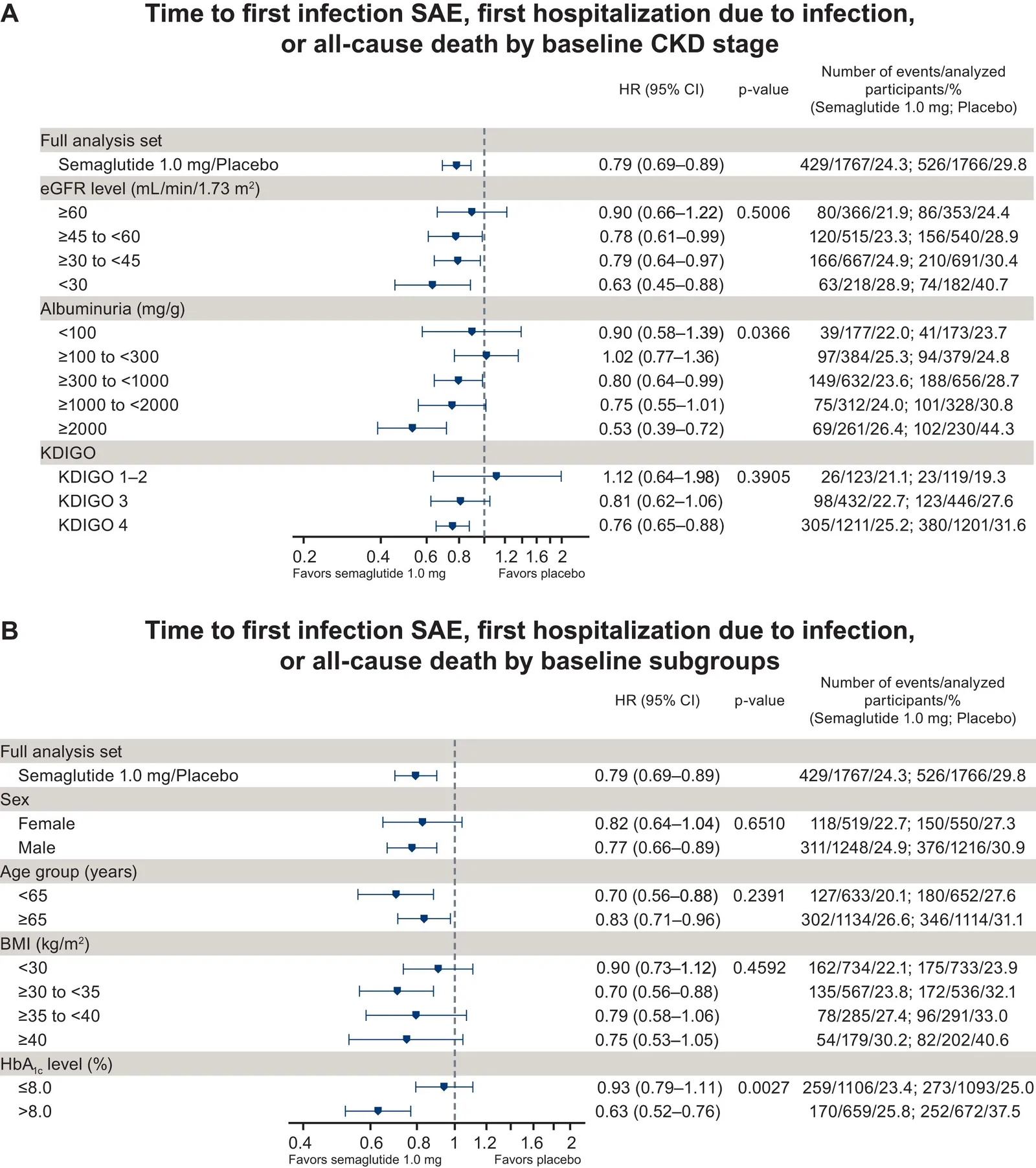
Time to main study outcome (first infection SAE, first hospitalization due to infection or all-cause death) by baseline CKD stage and participant characteristics. Data are from the in-trial period. Time from randomization to first infection SAE, first hospitalization due to infection or all-cause death by (**A**) baseline CKD stage and (**B**) other baseline characteristics.

The effect of semaglutide treatment on the main study outcome was consistent across participants with varying baseline eGFR levels and across major baseline subgroups by sex, age and BMI (Fig. 3). However, this risk reduction was greater in subgroups with baseline HbA_1c_ >8% [HR (95% CI) 0.63 (0.52–0.76)] versus ≤8% [HR (95% CI) 0.93 (0.79–1.11); *P_interaction_* = .0027] and baseline UACR ≥2000 mg/g [HR (95% CI) 0.53 (0.39–0.72)] versus UACR <100 mg/g [HR (95% CI) 0.90 (0.58–1.39); *P_interaction_*= .0367; Fig. 3].

**Figure 4:**
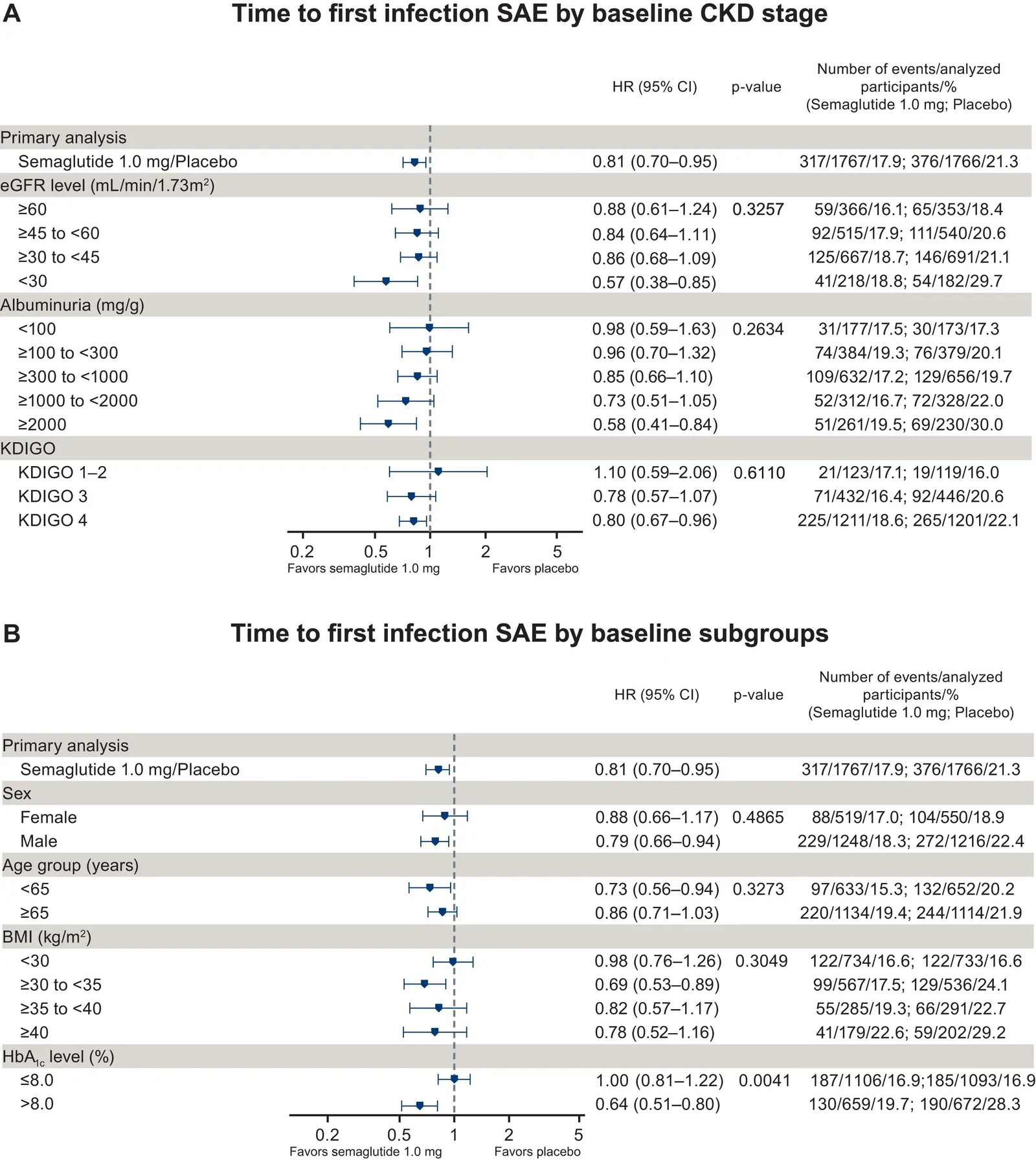
Time to first infection SAE by baseline CKD stage and participant characteristics. Data are from the in-trial period. Time from randomization to first infection SAE by (**A**) baseline CKD stage and (**B**) other baseline characteristics.

A similar pattern was observed for the overall incidence of infection SAEs (Fig. 4). The impact of semaglutide on infection SAEs was not significantly different in subgroups by sex, age or BMI at baseline, but there was an interaction for infection SAEs observed with baseline HbA_1c_ [HR (95% CI) 1.00 (0.81–1.22) for HbA_1c_ ≤8.0% versus 0.64 (0.51–0.80) for HbA_1c_ >8.0%; *P_interaction_* = .0041]. There was a non-significant difference in the HRs for infection SAEs in those with baseline UACR ≥2000 mg/g [HR (95% CI) 0.58 (0.41–0.84)] versus UACR <100 mg/g [HR (95% CI) 0.98 (0.59–1.63); *P_interaction_* = .2634]. The effect of semaglutide on the incidence of infection SAEs was unrelated to the degree of body weight loss during the trial (Supplementary data, Fig. S3).

**Figure 5:**
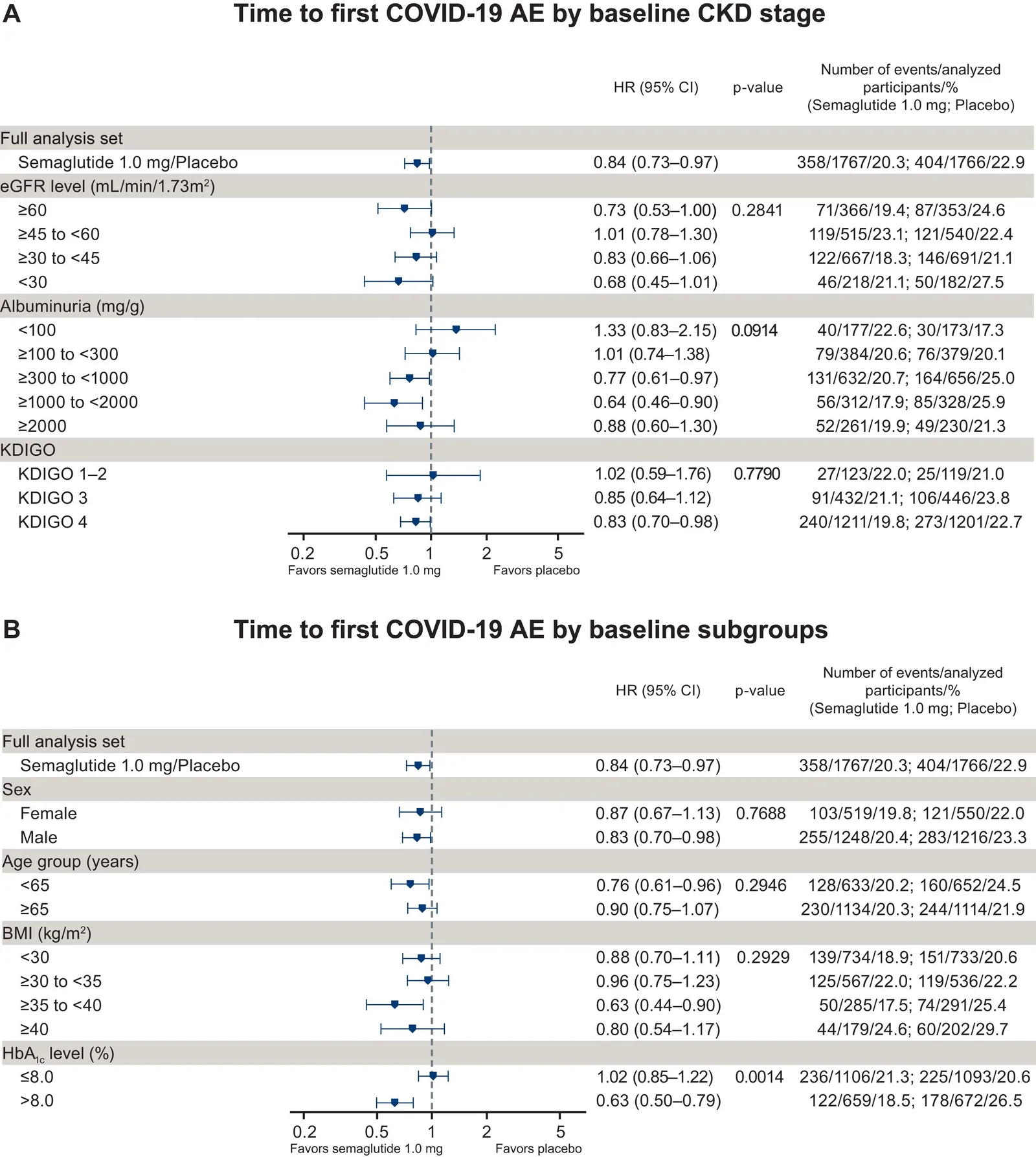
Time to first COVID-19 AE by baseline CKD stage and participant characteristics. Data are from the in-trial observation period. Time from randomization to first COVID-19 AE by (**A**) baseline CKD stage and (**B**) other baseline characteristics.

With regard to the incidence of COVID-19 AEs, the effect of semaglutide was significantly impacted by baseline HbA_1c_ [HR (95% CI) 1.02 (0.85–1.22) for HbA_1c_ ≤8.0% versus 0.63 (0.50–0.79) for HbA_1c_ >8.0%; *P_interaction_* = .0014; Fig. 5]. There was a numerically greater treatment effect on COVID-19 AE incidence in those with greater baseline UACR of ≥2000 mg/g [HR (95% CI) 0.88 (0.60–1.30)] versus UACR <100 mg/g [HR (95% CI) 1.33 (0.83–2.15); *P*_interaction_ = .0914].

## DISCUSSION

In the FLOW trial, treatment with once-weekly subcutaneous semaglutide 1.0 mg compared with placebo led to a 21% relative risk reduction for the main outcome, a composite of first infection SAE, first hospitalization due to infection or all-cause death. Relative risk of an infection SAE was reduced by 19% along with reduced risks of a COVID-19 AE by 16% and a COVID-19 SAE by 26%. Semaglutide treatment also resulted in a 17% decrease in the risk of hospitalization due to infection. The effect of semaglutide on infection SAEs was observed across sex, age and baseline BMI subgroups. However, interactions with baseline HbA_1c_ and UACR were observed, suggesting greater potential benefit of semaglutide on infection risk in those with poorer glycemic control and greater levels of albuminuria. Given the substantial incidence of serious infections including COVID-19 in FLOW, these data suggest that mitigation of infectious complications with semaglutide may provide a clinically important benefit in addition to improving kidney, CV and metabolic outcomes.

Respiratory infections are major complications in the CKD population. In the ASCEND-D (Anemia Studies in CKD: Erythropoiesis via a Novel prolyl hydroxylase inhibitor Daprodustat-Dialysis) and the ASCEND-ND (Aaemia Study in CKD: Erythropoiesis via a Novel prolyl hydroxylase inhibitor Daprodustat-Non-Dialysis) trials, which assessed treatment with daprodustat (a hypoxia-inducible factor prolyl hydroxylase inhibitor) in patients with CKD, infections accounted for 25%–30% of all deaths [20]. Moreover, co-occurrence of T2D and CKD further exacerbates the severity of infectious outcomes and increases risk of mortality [9, 21]. With regard to COVID-19, patients with T2D and CKD are more likely to experience severe outcomes such as hospitalization, acute kidney injury and death than those without, as well as higher overall healthcare utilization [22]. The present findings support a potential role for semaglutide to reduce risks of serious infections, COVID-19 events and related complications among high-risk participants with T2D and CKD. Additionally, a meta-analysis of 28 randomized controlled GLP-1 RA trials found a 14% reduction in the risk of contracting respiratory diseases in those treated with GLP-1 RAs compared with controls, with a risk reduction of 18% for semaglutide specifically [23]. Furthermore, in patients with T2D and CKD, treatment with GLP-1 RAs was associated with lower sepsis- and infection-related mortality when compared with dipeptidyl peptidase 4 (DPP-4) inhibitors [24]. Although the mechanisms are not fully understood, GLP-1 RAs may have potential anti-inflammatory or immunomodulatory effects [23]. In a non-diabetic mouse model of sepsis, GLP-1 RAs reduced the systemic inflammatory response via central nervous system GLP-1 receptor activation, as reflected by lower levels of circulating tumor necrosis factor-α, reduced Toll-like receptor activity in various cells and tissues, and lessened acute lung injury [25]. GLP-1-based therapies may have anti-inflammatory effects in people with chronic inflammatory diseases [26].

The SELECT (Semaglutide Effects on Cardiovascular Outcomes in People with Overweight or Obesity) trial, which evaluated the efficacy of once-weekly subcutaneous semaglutide 2.4 mg versus placebo in participants with obesity and CVD but without diabetes, also demonstrated an overall reduction in rates of infection, as well as infection-related death and COVID-19-related death [27]. Although corresponding mortality benefits were not demonstrated in the present study, this result may be related to the higher dose of semaglutide used in SELECT (2.4 mg weekly) versus FLOW (1 mg weekly), different study populations, or the lower statistical power in FLOW due to a smaller sample size.

Subgroup analyses revealed greater benefit of semaglutide on the main infection outcome and infection SAEs in participants with high levels of HbA_1c_ or albuminuria at baseline. Some of these benefits of semaglutide may be related to the lowering of glycemia or albuminuria over the course of the trial, particularly in study participants starting with poorer glycemic control or more severe CKD. There was a numerical difference in baseline HbA_1c_ observed in the placebo group between participants who had an infection/COVID-19 during the trial and those who did not; this difference was not seen in the semaglutide group. Importantly, baseline differences in the placebo group do not establish HbA_1c_ as a predictor of risk of infection or COVID-19, and higher infection event rates in the placebo group versus semaglutide may not be attributed to these baseline differences. Future studies should further explore the mechanisms behind these interactions and potential impact on considerations for use of semaglutide.

### Limitations

Although the FLOW trial enrolled a large population of adults with T2D and CKD, with a relatively long follow-up period, the present analyses had limitations. These results are applicable to patients meeting the FLOW trial inclusion criteria, and thus, 93% had high-to-very-high–risk CKD according to Kidney Disease: Improving Global Outcomes (KDIGO) criteria [2]. The applicability of these findings to a CKD population with lower risk remains to be determined. Moreover, in this analysis, infections were only documented if they met the prespecified criteria for an SAE; therefore, most of the outcomes in this study capture infections (including COVID-19) with serious clinical manifestation, excluding virus-positive cases with mild (or no) symptoms. Although nearly three-quarters of FLOW participants were vaccinated for COVID-19, different types of vaccine were administered at various times before and during this global trial, which precludes assessment of how vaccines may have influenced infection-related or other outcomes. Due to pragmatic and logistical constraints during the pandemic, the diagnosis of COVID-19 in the FLOW trial was often based on clinical criteria, which could have introduced an element of misclassification. Finally, the FLOW trial was not powered for subgroup analyses.

## CONCLUSIONS

Semaglutide reduced the composite risk of infection SAEs, hospitalization due to infection, and all-cause death, and lowered rates of COVID-19 AEs and SAEs in FLOW trial participants with T2D and CKD. In addition to improving kidney, CV and metabolic outcomes, semaglutide may reduce risk of infections in this population.

## Supplementary Material

gfag036_Supplemental_File

## Data Availability

An authorised researcher can request access to clinical trial study data by submitting a research proposal for review and approval by Novo Nordisk and an internal independent review panel. Requests are usually considered after the research is finished and the main results have been published. If the research supports a regulatory application, requests will be considered after the product and its intended use are approved in both the EU and the USA. Participants’ clinical data will be anonymised, following an approved internal process, before data are shared to external third parties. For details on how to request access to clinical data, visit novonordisk-trials.com.
