# Supplementary material for: Effect of semaglutide on COVID-19 and other infections: an analysis from the FLOW randomized clinical trial
Source: Nephrol Dial Transplant. 2026 Feb 23;41(9):1696–708. doi: 10.1093/ndt/gfag036 (PMC13518088; doi:10.1093/ndt/gfag036)
Supplement: Supplementary file 1 [file gfag036_supplemental_file.docx]

**SUPPLEMENTAL ONLINE CONTENT**

Rayner B, et al. Effect of semaglutide on COVID-19 and other infections: An analysis from the FLOW randomized clinical trial. *Nephrol Dial Transplant.* Published online [placeholder for date and DOI].

**Contents**

[Supplementary methods	2](#_Toc219303685)

[Supplementary Table S1. COVID-19 vaccines initiated in participants who experienced a COVID-19 AE during the in-trial period	3](#_Toc219303686)

[Supplementary Table S2. COVID-19 vaccines initiated in participants who did not experience a COVID-19 AE during the in-trial period	5](#_Toc219303687)

[Supplementary Figure S1. Most common infection SAEs reported during the trial.	8](#_Toc219303688)

[Supplementary Figure S2. Incidence of most common acute kidney failure events by preferred term in participants with and without a COVID-19 AE.	9](#_Toc219303689)

[Supplementary Figure S3. SAEs by body weight loss at week 104.	10](#_Toc219303690)

# SUPPLEMENTARY METHODS

**Classification of infection SAEs and hospitalizations due to infection**

Infection SAEs and hospitalizations due to infections could be recorded separately; this is because AE data were collected via a specific form which allowed investigators to specify whether an AE was serious and/or whether the AE resulted in hospitalization. Categorization of infection AEs as SAEs or hospitalizations due to infection was not related to whether the AE was due to a COVID-19 infection.

# Supplementary Table S1. COVID-19 vaccines initiated in participants who experienced a COVID-19 AE during the in-trial period.

| **Vaccine** | **Semaglutide 1.0 mg**  (n=358) | **Placebo**  (n=404) | **Total**  (n=762) |
| --- | --- | --- | --- |
| **COVID-19 vaccines** | 269 (75.1) | 286 (70.8) | 555 (72.8) |
| Pfizer BioNTech COVID-19 Vaccine | 124 (34.6) | 131 (32.4) | 255 (33.5) |
| COVID-19 Vaccine | 84 (23.5) | 78 (19.3) | 162 (21.3) |
| Moderna COVID-19 Vaccine | 52 (14.5) | 56 (13.9) | 108 (14.2) |
| Comirnaty | 48 (13.4) | 42 (10.4) | 90 (11.8) |
| AstraZeneca COVID-19 Vaccine | 19 (5.3) | 32 (7.9) | 51 (6.7) |
| COVID-19 Vaccine AstraZeneca | 17 (4.7) | 26 (6.4) | 43 (5.6) |
| Spikevax | 6 (1.7) | 15 (3.7) | 21 (2.8) |
| COVID-19 Vaccine Moderna | 9 (2.5) | 11 (2.7) | 20 (2.6) |
| Sputnik V | 7 (2.0) | 11 (2.7) | 18 (2.4) |
| CoronaVac | 8 (2.2) | 5 (1.2) | 13 (1.7) |
| Vaxzevria | 5 (1.4) | 4 (1.0) | 9 (1.2) |
| Gam-COVID-Vac | 3 (0.8) | 6 (1.5) | 9 (1.2) |
| Covishield | 4 (1.1) | 5 (1.2) | 9 (1.2) |
| Sputnik Light | 1 (0.3) | 1 (0.2) | 2 (0.3) |
| Covaxin (COVID-19 Vaccine Inact W.Virion NIV-2020-770) | 1 (0.3) | 1 (0.2) | 2 (0.3) |
| Spikevax Bivalent Original/Omicron Ba.1 | 1 (0.3) | 0 | 1 (0.1) |
| Moderna COVID-19 Vaccine, Bivalent (Original and Omicron Ba.4/Ba.5) | 0 | 1 (0.2) | 1 (0.1) |
| Janssen COVID-19 Vaccine | 0 | 1 (0.2) | 1 (0.1) |
| COVID-19 Vaccine Janssen | 0 | 1 (0.2) | 1 (0.1) |
| COVID-19 Vaccine (Vero Cell), Inactivated (COVID-19 Vaccine Inact [Vero] WIV04) | 1 (0.3) | 0 | 1 (0.1) |

Values are no. (%). Vaccine names reflect what was recorded by the reporting investigator and may include both brand and generic names; thus, entries are not consolidated and may represent the same product under different names.

AE, adverse event; COVID-19, coronavirus disease-2019.

# Supplementary Table S2. COVID-19 vaccines initiated in participants who did not experience a COVID-19 AE during the in-trial period.

| **Vaccine** | **Semaglutide 1.0 mg**  (n=1412) | **Placebo**  (n=1365) | **Total**  (n=2777) |
| --- | --- | --- | --- |
| **COVID-19 vaccines** | 1047 (74.2) | 997 (73.0) | 2044 (73.6) |
| Pfizer BioNTech COVID-19 Vaccine | 442 (31.3) | 453 (33.2) | 895 (32.2) |
| COVID-19 Vaccine | 248 (17.6) | 251 (18.4) | 499 (18.0) |
| Moderna COVID-19 Vaccine | 193 (13.7) | 207 (15.2) | 400 (14.4) |
| Comirnaty | 144 (10.2) | 125 (9.2) | 269 (9.7) |
| AstraZeneca COVID-19 Vaccine | 89 (6.3) | 85 (6.2) | 174 (6.3) |
| COVID-19 Vaccine AstraZeneca | 72 (5.1) | 66 (4.8) | 138 (5.0) |
| Covishield | 72 (5.1) | 61 (4.5) | 133 (4.8) |
| Sputnik V | 51 (3.6) | 40 (2.9) | 91 (3.3) |
| CoronaVac | 40 (2.8) | 28 (2.1) | 68 (2.4) |
| Spikevax | 33 (2.3) | 19 (1.4) | 52 (1.9) |
| COVID-19 Vaccine Moderna | 23 (1.6) | 17 (1.2) | 40 (1.4) |
| Gam-COVID-Vac | 24 (1.7) | 15 (1.1) | 39 (1.4) |
| Covaxin (COVID-19 Vaccine Inact W.Virion NIV-2020-770) | 19 (1.3) | 6 (0.4) | 25 (0.9) |
| Vaxzevria | 13 (0.9) | 10 (0.7) | 23 (0.8) |
| Sputnik Light | 9 (0.6) | 4 (0.3) | 13 (0.5) |
| Comirnaty Original/Omicron Ba.4-5 | 3 (0.2) | 4 (0.3) | 7 (0.3) |
| Janssen COVID-19 Vaccine | 2 (0.1) | 3 (0.2) | 5 (0.2) |
| mRna-1273 | 1 (<0.1) | 3 (0.2) | 4 (0.1) |
| Gam-COVID-Vac Component 1 | 2 (0.1) | 1 (<0.1) | 3 (0.1) |
| COVID-19 Vaccine (Vero Cell), Inactivated (COVID-19 Vaccine Inact [Vero] HB02) | 3 (0.2) | 0 | 3 (0.1) |
| Tozinameran | 2 (0.1) | 0 | 2 (<0.1) |
| Gam-COVID-Vac Component 2 | 1 (<0.1) | 1 (<0.1) | 2 (<0.1) |
| Comirnaty Bivalent Ba.4/Ba.5 | 1 (<0.1) | 1 (<0.1) | 2 (<0.1) |
| VidPrevtyn Beta | 0 | 1 (<0.1) | 1 (<0.1) |
| Vacuna COVID-19 Pfizer BioNTech | 1 (<0.1) | 0 | 1 (<0.1) |
| Sputnik V Component 2 | 0 | 1 (<0.1) | 1 (<0.1) |
| SARS-CoV-2 Vaccine | 1 (<0.1) | 0 | 1 (<0.1) |
| CoviVac | 1 (<0.1) | 0 | 1 (<0.1) |
| COVID-19 Vaccine NRVV Ad26 (Gam-COVID-Vac) | 1 (<0.1) | 0 | 1 (<0.1) |
| COVID-19 Vaccine Janssen | 1 (<0.1) | 0 | 1 (<0.1) |
| COVID-19 Vaccine (Vero Cell), Inactivated (COVID-19 Vaccine Inact [Vero]) | 1 (<0.1) | 0 | 1 (<0.1) |
| COVID-19 Vaccine (Vero Cell), Inactivated (COVID-19 Vaccine Inact [Vero] CZ02) | 1 (<0.1) | 0 | 1 (<0.1) |
| ChAdOx1 nCoV-19 | 1 (<0.1) | 0 | 1 (<0.1) |
| Abdala | 1 (<0.1) | 0 | 1 (<0.1) |

Values are no. (%). Vaccine names reflect what was recorded by the reporting investigator and may include both brand and generic names; thus, entries are not consolidated and may represent the same product under different names.

AE, adverse event; COVID-19, coronavirus disease-2019.

# Supplementary Figure S1. Most common infection SAEs reported during the trial.

Shown are infection and infestation SAEs (at the preferred-term level) reported in ≥0.5% of either treatment group during the trial. SAEs are sorted in descending order at the preferred-term level by occurrence in the semaglutide 1.0 mg group.

COVID-19, coronavirus disease-2019; SAE, serious adverse event.

# Supplementary Figure S2. Incidence of most common acute kidney failure events by preferred term in participants with and without a COVID-19 AE.

Plot shows incidence of most common acute kidney failure events in those with and without a COVID-19 AE, where absolute count is >1 in the total population. Preferred terms are sorted in descending order by occurrence in the semaglutide 1.0 mg group.

AE, adverse event; COVID-19, coronavirus disease-2019.

# Supplementary Figure S3. SAEs by body weight loss at week 104.

Plot shows incidence of the 10 most common SAEs (at the system organ class level) by degree of body weight loss at week 104, where absolute count is >1 in the total population. System organ class events are sorted in descending order of occurrence in the semaglutide 1.0 mg group. Participants still in the trial at week 104 but with missing body weight measurement had their body weight imputed by multiple imputation. The multiple imputation model included baseline value as a covariate and use of SGLT2is (yes/no) at baseline as a fixed factor and was fitted to all participants with a measurement regardless of treatment status at week 104. The fitted model was used to impute values for participants with missing data at week 104. Body weight loss for each participant was calculated as the mean of the 500 imputations. Participants who left the trial or died before week 104 had their missing values imputed by the treatment group mean.

BW, body weight; SAE, serious adverse event; SGLT2i, sodium–glucose cotransporter-2 inhibitor.
